# Using superparamagnetic iron oxide nanoparticles to enhance bioavailability of quercetin in the intact rat brain

**DOI:** 10.1186/s40360-018-0249-7

**Published:** 2018-09-25

**Authors:** Rezvan Enteshari Najafabadi, Nasrin Kazemipour, Abolghasem Esmaeili, Siamak Beheshti, Saeed Nazifi

**Affiliations:** 1 Department of Basic Sciences, School of Veterinary Medicine, Shiraz University, Shiraz, Iran; 2 Cell, Molecular Biology and Biochemistry Division, Department of Biology, Faculty of Sciences, University of Isfahan, P.O. Box: 8174673441, Isfahan, Iran; 3 Department of Clinical Science, School of Veterinary Medicine, Shiraz University, Shiraz, Iran

**Keywords:** Oral drug delivery, HPLC, Nanoparticles, Quercetin, SPION

## Abstract

**Background:**

Quercetin (QT) as a bioactive flavonoid has a potential therapeutic activity for numerous neuronal injuries and neurodegenerative diseases. However, the low absorption rate of QT, especially through the blood-brain barrier, restricts its bioactivity in the body. The current research took the advantage of superparamagnetic iron oxide nanoparticles (SPIONs) to enhance the bioavailability of quercetin.

**Methods:**

Quercetin conjugated with SPIONs was prepared by means of nanoprecipitation method and was characterized by X-ray diffractometer, scanning electron microscope, and Fourier transformed infrared spectrometer analyses. Wistar male rats were orally fed by gavage with QT and QT-SPION at 50 and 100 mg/kg daily doses for 7 days. Using high-performance liquid chromatography (HPLC) method, biodistribution of QT was evaluated in plasma and brain tissue.

**Results:**

The outcomes of this research revealed a higher concentration in the plasma and brain of the rats fed with QT-SPION in comparison to free QT.

**Conclusion:**

The results of this study confirm that SPION as a targeted drug delivery system enhances the bioavailability of quercetin in the brain about ten folds higher than free quercetin and could be used for the treatment of neurodegenerative disorders.

## Background

Nanoparticles (NPs) are demarcated as particles up to 1000 nm especially less than 200 nm in diameter. NPs as capable vehicles have been applied to deliver some drugs and reagents into the numerous tissues and organs including the central nervous system (CNS). Superparamagnetic iron oxide nanoparticles (SPIONs, Fe_3_O_4_ NPs) is one of the most interesting NPs. These nanoparticles have magnetic properties and, therefore, using an external magnet, SPION could be used as a targeted drug delivery system. Also, SPIONs are considered for cell tracking, bioseparation, magnetic resonance imaging (MRI), and magnetic hyperthermia [1, 2].

Iron ions may release after utilization of iron oxide nanoparticles and accumulate in the body tissue [3]. This depends on the size, concentration, surface charge, and the type of coating and side-groups of iron oxide nanoparticle. Iron ions through oxidative stress may lead to macromolecule damage [3]. Accordingly, the application of iron oxide nanoparticles in the central nervous system needs serious care [4].

Several neurodegenerative disorders, such as Alzheimer’s disease, affect the structure and function of the CNS. In spite of the gradually growing of drugs for the treatment of CNS disorders, there are few potential novel drugs available [5–7]. This is because of poor bioavailability and also the failure of many therapeutic molecules to cross the blood-brain barrier (BBB) [8]. Instead, any drug entry into the brain via parenteral administration is severely controlled by the BBB. A report suggests that over 98 % of small molecules and almost all the large molecules cannot penetrate to the brain [9].

It has been attempted to increase the bioavailability of the therapeutic molecules. One approach is increasing the concentration of drug in blood circulation by nanoparticles. Another approach is increasing the permeability of the BBB. Strategies used to increase BBB permeability are categorized as invasive and non-invasive. The first strategy includes the momentary enhancement of the BBB permeability [10], agent intracerebral grafting [11], or direct infusion of the drug via intraventricular or intracerebral routes [12]. The invasive methods are expensive and are associated with problems, such as brain infections or edema [13]. The non-invasive strategies comprise an alteration of the drug molecules by means of chemical [14] or biochemical methods [15], accompanied by means of the olfactory route [16]. After modification, they may have certain disadvantages such as losing drug activity. As mentioned above the utilization of nanoparticles especially SPIONs could overcome these limitations.

Quercetin (3,3′,4′,5,7-pentahydroxyflavone, (QT)) is an active biological flavonoid found in large quantities in edible fruits, vegetables and medicinal plants such as onions, cabbage, and apples [17–19]. Quercetin has antiinflammatory [20], antioxidant [21], anti-cancer [22], anti-viral [23], and anti-ischemic effects [24, 25]. Also, quercetin may alter glucose homeostasis in the brain [26]. Neuronal protection of QT has been reported in both in vitro and in vivo investigations [27–31]. Therefore, QT has a potential therapeutic for various neuronal injuries and neurodegenerative diseases [19]. However, the solubility of QT in water is low and it has a short biological half-life. Also, it has poor oral and CNS bioavailability. Accordingly, these limitations may overcome by means of the SPIONs [32–34]. Consequently, Kumar et al. and Akal et al. [35, 36] conjugated quercetin with SPIONs. Both studies used an in vitro method to evaluate the effect of quercetin conjugated with SPIONs on cells. None of them did investigate the bioavailability of quercetin-SPIONs in vivo.

Therefore, in the current study, the quercetin conjugated dextran coated Fe_3_O_4_ nanoparticles (QT- Fe_3_O_4_ NPs) were prepared by chemical precipitation method and the quercetin bioavailability enhancement in an experimental rat model was investigated.

In this requirement, Wistar rats were used because they are of the same purebred species. In addition, rats are used as models in medical testing due to their genetic, biological and behaviour characteristics closely resemble those of humans, and many symptoms of human conditions can be replicated in rats.

## Methods

No additional purification was performed on reagents used in this study. The chemicals were bought from Fluka, Merck, and Sigma–Aldrich chemical companies.

Synthesis of quercetin conjugated iron oxide nanoparticles (QT-SPION).

Quercetin conjugated Fe_3_O_4_ nanoparticles were prepared as Kumar et al. described in 2014 with some modifications [35]. First, chemical coprecipitation method was used to synthesize dextran coated Fe_3_O_4_ nanoparticles. Briefly, 1.135 g FeCl_3_ anhydrous, 0.695 g FeCl_2_ and 0.45 g dextran were mixed and dissolved in 200 ml deionized (DI) water. The chemical reaction is as follow: Fe (II) + 2Fe (III) + 8OH → Fe3O4 + 4H2O. The mixture was then placed into a three-neck flask equipped with a mechanical stirrer. After complete mixing, ammonia solution was dropped into the mixture under argon protection with vigorous stirring. This operation was continued until the solution reached a pH of 9. Then the solution was kept at 90 °C for 2 h with constant moving. A strong magnet was applied to collect the subsequent precipitate. Then using DI water and ethanol the supernatant was washed several times. To dry out the supernatant, it was placed into an oven at 70 °C overnight.

QT-SPION nanoparticles were prepared by addition of QT to dextran-coated Fe_3_O_4_ nanoparticles. For this purpose, the pre-determined amount of 1-ethyl-3[3-dimethylaminopropyl] carbodiimide hydrochloride (EDC) and N-hydroxysuccinimide (NHS) was added to the dextran-coated Fe_3_O_4_ nanoparticles under ultrasonication according to related protocols. Then, under ultrasonication, a required amount of quercetin in dimethyl sulfoxide (DMSO) solution was added to the mixture and was stirred for 24 h at room temperature. To eliminate any free drug or any other biological scums, the precipitate was washed with DMSO and acetone. Then, the precipitate was collected using a strong external magnet and was dried out by means of a freeze drier.

### Characterization techniques

Fourier transformed infrared (FTIR) spectroscopy was recorded on a Jasco 6300 spectrophotometer in the range of 400 and 4000 cm^− 1^ by KBr pellet method. X-ray diffractometer (XRD) patterns of magnetite nanoparticles were carried out in a PANalytical X’PERT PRO powder X-ray diffractometer with Cu Kα_1_ (k = 1.54056 Å) radiation at room temperature. Hitachi S–4700 field emission–scanning electron microscope (FE–SEM), equipped with an energy-dispersive X-ray analysis (EDX) detector was used to obtain the morphological features of nanoparticles.

### Drug release study and loading

To measure the in vitro release rate of QT from QT-SPIONs in phosphate buffer saline (PBS) medium a dialysis bag method was used. Briefly, by means of cellulose dialysis tube bags, 1 mg of QT (QT-SPIONs) was suspended in 1.5 mL of PBS (pH 7.4). The dialysis with a magnetic stirrer was performed at 37 °C. Two ml of the sample was picked up at regular time intervals for 20 h. To measure the amount of QT in the medium spectrophotometer at 374 nm wavelength was used.

Cumulative percentage quercetin release from QT-SPIONs, into PBS with pH values of 7.4 was calculated using the below equation,

$$ \mathrm{Cumulative}\ \mathrm{percentage}\ \mathrm{drug}\ \mathrm{release}\ \left(\%\right)=\mathrm{Volume}\ \mathrm{of}\ \mathrm{sample}\ \mathrm{withdrawn}\ \left(\mathrm{ml}\right)/\mathrm{bath}\ \mathrm{volume}\ \left(\mathrm{v}\right)\times \mathrm{P}\ \left(\mathrm{t}-1\right)+\mathrm{Pt} $$

Where Pt is % release at time t and P (t – 1) is % release prior to ‘t’. The results were shown as mean ± SEM of triplicate experiments.

To measure quercetin loading in quercetin conjugate with iron oxide nanoparticles, the amount of quercetin was measured using a spectrophotometer at 260 nm and then, according to the amount of quercetin consumed, loading was calculated.

### Animals

Adult male Wistar rats (*n* = 42), weighing between 180 and 200 g, were bought from Royan Institute (Isfahan, Iran). All experiments were carried out in accord with the guide for the care and use of laboratory animals (USA National Institute of Health publication No. 80–23, revised 1996). Also, all procedures were approved by the graduate studies committees of the University of Isfahan and Shiraz University. Rats were randomly divided into groups of 6 rats and were housed 3/cage in a room with a temperature of 25 °C ± 2 °C and a 12 h light/dark cycle (lights on at 07:00 a.m.). All animals had free access to tap water and diet for one week before the experiment. Not any problem was detected during the experimental procedures. Sample sizes were based on Mead’s resource equation. Based on this formula the optimum size of an experiment usually has between 10 and 20 error degrees of freedom (E). In calculating the number of rats needed, the probable casualties were also considered.

### Dosing and sampling

All of the treatments (QT, SPION, and QT-SPION) were dissolved in deionized water (DI) and gavaged (08:00–10:00 am) in separate groups of rats. Rats in treatment groups received 50 or 100 mg/kg of body weight QT, SPION, or QT-SPION.

Short-term feeding: Quercetin conjugated Fe_3_O_4_ nanoparticles (QT-SPIONs) were freshly prepared for ingesting administration via gavage. Group one was treated with a dose of free QT solution (100 mg/kg). Group two was administered with a dose of 100 mg/kg of QT-SPIONs. Then 3, 6, 9, 12, and 24 h later, blood samples (almost 1 ml) were collected from the tail vein and poured into centrifuge tubes containing heparin. Then, plasma was obtained via centrifugation at 4 °C (3000×g, 10 min) and kept at − 70 °C till use.

Long-term feeding: Rats in this test were divided into five groups. Group one and two were administered with 50 mg/kg and 100 mg/kg free QT solution, respectively. Group three received 50 mg/kg QT-SPIONs and group four treated with 100 mg/kg QT-SPIONs. All constructions were administered orally at a daily dose for a period of 7 days. Group five served as control and received neither QT nor QT-SPIONs. Twenty-four hours after of the last feeding each rat was anesthetized by intraperitoneal injection of sodium pentobarbital. Then the blood (1 ml for each rat) was taken from the heart and was put into tubes containing heparin. Plasma was collected as described above and stored at − 70 °C until use. To determine the distribution of the quercetin in the brain, rats were sacrificed after administration of a mixture of ketamine (100 mg/kg, i.p) and xylazine (10 mg/kg, i.p) anaesthesia. Samples of the brains were rapidly removed and kept at − 70 °C before the examination.

### Preparation of plasma and brain samples

A 100 μl of plasma was supplemented with one ml of methanol, and the combination was vortexed for five minutes and then centrifuged (10,000 rpm, Eppendorf, 5417R, USA) for 10 min at 4 °C. The collected clear supernatant was filtered using 0.20 μm syringe filters. To measure QT in plasma, 20 μl of the sample was injected into the HPLC system. Approximately 100 mg of brain tissue was homogenized in 500 μl in 1× PBS (pH 7.4). Then, the homogenates were centrifuged at 10,000 rpm for 10 min at 4 °C. Then, 300 μl of methanol with 200 μl aliquots of the clear tissue homogenates were added and the scattering was vortexed for two minutes. Then the samples were centrifuged at 10,000 rpm for 10 min at 4 °C. The supernatant was filtered using 0.20 μm syringe filters and 20 μl of the brain sample was injected into the HPLC system to measure QT.

### HPLC analysis of quercetin

HPLC was performed as previously described [19]. Briefly, quercetin was evaluated by HPLC equipped with a reverse-phase C18 column (150 mm × 4.6 mm, pore size 5 μm, ProntoSIL), a UV detector and a vacuum degasser run by Smartline-2600 software. Methanol and water (70:30 *v*/v) (pH adjusted to 3.64 with glacial acetic acid) was used as the mobile phase. To separate QT, an isocratic condition with column temperature of 25 °C, a constant flow rate of 1.0 ml/min, 20 μl injection volume and a detection wavelength of 374 nm, was performed. To evaluate the linear relationship between the peak areas and the concentrations of quercetin the calibration curves were generated. The coefficient of determination (r^2^), slope, and intercept were calculated as regression parameters by weighted linear regression.

### Iron determination

Plasma and brain samples were prepared as described above. The iron concentrations of plasma (at the time point of 3, 6, 9, 12, and 24) and brain tissue (24 h after oral administration of QT-SPIONs) were measured by ICP-OES (inductively coupled plasma-optical emission spectroscopy). To assay iron concentration, one gram of the sample was mixed with 1 ml sulphuric acid (98%) in a tube and heated at 100 °C for 5 min. Every ten minutes 3 ml of perchloric acid was added to the tube and heated for 40 min. After digestion, the solution was diluted to with 10 ml of distilled water for ICP-OES tests.

### Liver histology

At the end of the study to investigate histopathologic alterations, animals were killed by ketamine anesthesia. Liver samples of control rats and animals treated with 100 mg/kg of free QT, SPION and QT-SPION were removed and fixed instantaneously in 10% formalin in order to routine paraffin embedding. Paraffin blocks with 3 mm thickness were prepared and stained with haematoxylin–eosin (H–E) method. Finally, sections were examined under Leitz microscope at 400_ magnification.

### Statistical analysis

The results were reported as mean ± SEM (standard error mean) for the six independent experiments. Oneway ANOVA followed by a multiple comparisons Tukey’s test or Student’s t-test was used to estimate the difference as multiple comparison tests. For these procedures, *GraphPad Prism* software version 6 was used. For all analyses, *P* values of less than of 0.05 were considered statistically significant.

## Results

### Physicochemical characterization of QT-SPIONs

**Fig. 1:**
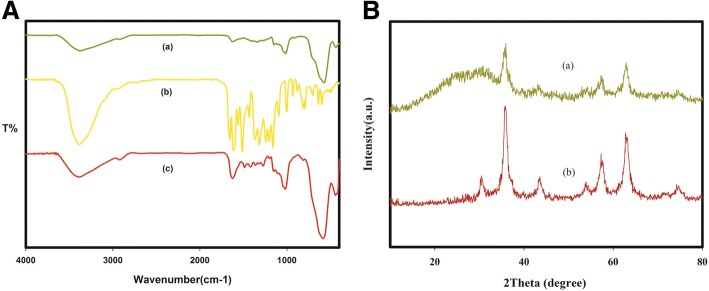
FTIR and XRD profiles. **a** Comparison of FTIR spectra of dextran coated iron oxide nanoparticle, pure quercetin and quercetin conjugated Fe_3_O_4_ nanoparticles. The samples fro top to bottom are (a) dextran coated SPIONs and (b) pure quercetin and (c) quercetin conjugated Fe_3_O_4_ nanoparticles. **b** XRD pattern for the (a) dextran coated and (b) quercetin conjugated Fe_3_O_4_ nanoparticles

The FTIR spectrum of dextran-coated Fe_3_O_4_ nanoparticles, quercetin, and quercetin conjugated magnetite nanoparticles (QT-SPIONs) are shown in Fig. 1A (a–c). The spectrum of dextran-coated Fe_3_O_4_ nanoparticle (Fig. 1A (a)) displayed a durable absorption band at 573 cm^− 1^ that was allocated to the Fe–O vibration frequency of magnetite spinel structure and the broad peaks at 3377 cm^− 1^ represent the OH stretching vibration of the hydroxyl groups. FTIR of quercetin (Fig. 1A (b)) showed characteristic bands corresponding to OH groups at 3388 cm^− 1^, to C=O absorption at 1656 cm^− 1^ and a region corresponding to C-O stretching at 1150–1070 cm^− 1^. Also, the peak 931 cm^− 1^ represents the C-H bending vibration of aromatic groups. The spectrum of the synthesized QT-SPIONs (Fig. 1A (c)) showed broad absorption bands at 3386 cm^− 1^, which were assigned to the stretching vibrations of hydroxyl groups and other characteristic bands that confirmed the successful conjugation of quercetin on the Fe_3_O_4_ nanoparticles.

The crystalline structure and phase analysis of magnetite nanoparticles were examined by XRD analysis. The pattern of powder XRD for the synthesized magnetic nanoparticles was close to that of crystalline magnetite Fe_3_O_4_ (Fig. 1B). In addition, the characteristic peaks of Fe_3_O_4_ NPs at 30.1°, 35.4°, 43.9°, 53.4°, 57.0° and 62.6° were detected for dextran coated and quercetin conjugated Fe_3_O_4_ NPs approving the existence of the crystalline structure of the magnetite.

**Fig. 2:**
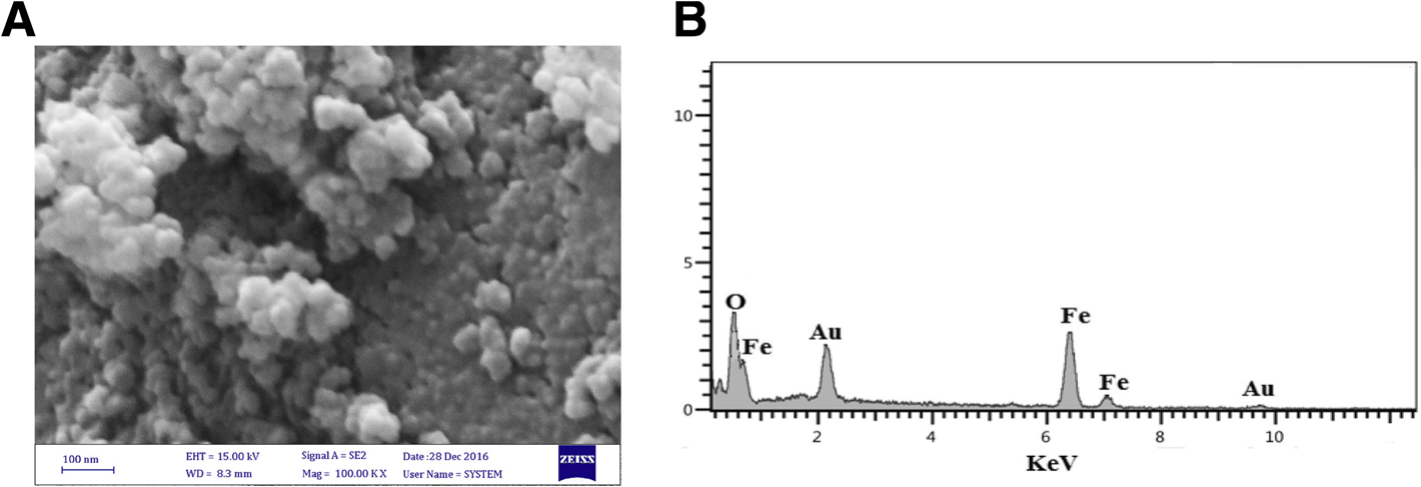
**a** SEM image of quercetin conjugated Fe_3_O_4_ nanoparticles. **b** SEM-EDX spectrum of quercetin conjugated Fe_3_O_4_ nanoparticles

The surface morphology and size of the quercetin conjugated Fe_3_O_4_ nanoparticles obtained were directly visualized by FE-SEM (Fig. 2a). As can be seen, the nanoparticles are spherical and have diameters in the range of 30 to 50 nm. The presence of the iron and oxygen was also confirmed by the EDX detector coupled to the SEM (Fig. 2b).

**Fig. 3:**
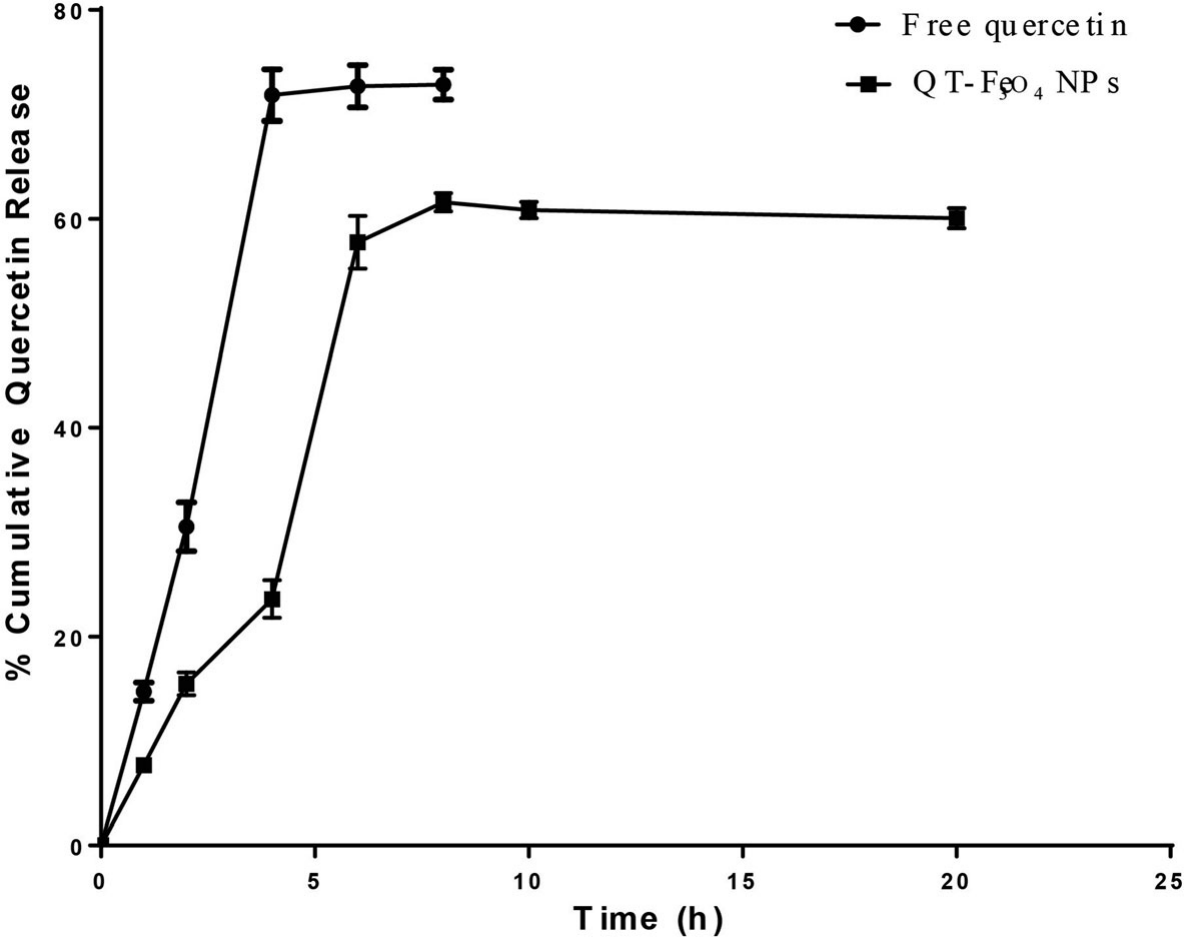
In vitro cumulative percent drug release versus time profile of quercetin. Plot showing percent cumulative release of QT from QT-Fe_3_O_4_ NPs in comparison with the free QT in pH 7.4 phosphate-buffered saline (PBS). Results are presented as mean ± SEM (*n* = 3). h, hours

The release rate of quercetin from either solution containing free quercetin or solution inclosing QT-SPION was primarily rapid and then it was slower as time continued. In the beginning, 71.8 ± 2.5% and 23.5 ± 1.8% of the drug was released in 4 h from free quercetin and QT-SPION respectively. This was progressively amplified and turn out to be a maximum value of 61.6 ± 0.8% in 8 h for quercetin released from QT-SPION. The release rate of QT from NPs was slower and more constant at the rate of around 60% over the period of 20 h (Fig. 3). These results showed that conjugation of quercetin with SPION decreased the release of QT from the NPs compared to that free from of QT.

To calculate quercetin loading, the difference in quercetin concentration used in the preparation of quercetin conjugate and the concentration of quercetin in the subfiltration solution was calculated and the quercetin concentration was used. The percentage of quercetin loading was 42%.

### Detection of quercetin in plasma

**Fig. 4:**
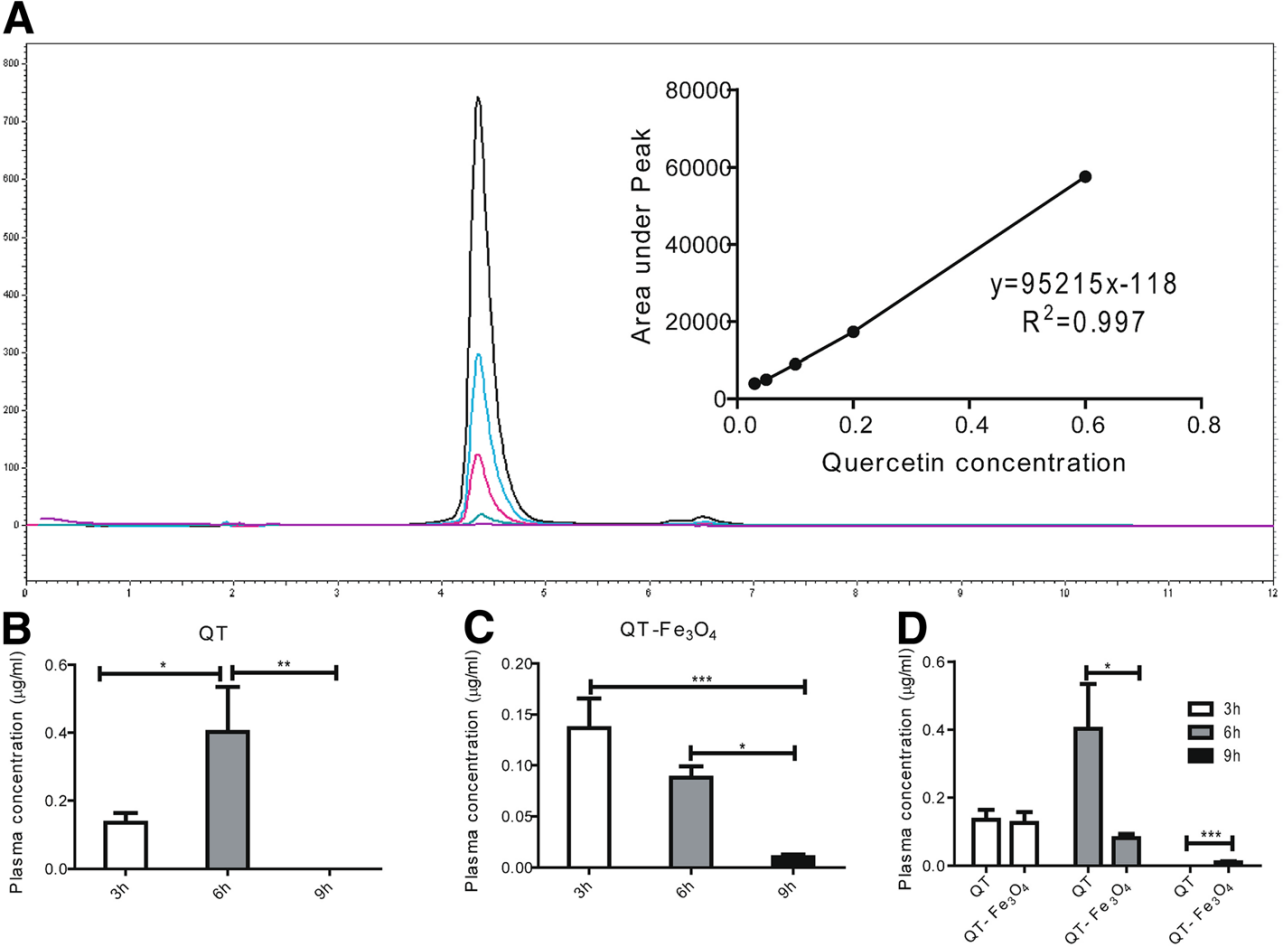
HPLC analyses for the identification of quercetin in rat plasma after oral administration of quercetin and quercetin conjugated Fe3O4 nanoparticles in Wistar rats. **a** Quercetin standards. Different serial dilutions are shown: 0.6 μg/ml (black), 0.2 μg/ml (blue), 0.1 μg/ml (red), and 0.05 μg/ml (green). The inset shows the calibration curve (Peak area vs. Concentration). **b** free QT, and **c** QT-SPIONs, and **d** comparison of the concentration of free QT with QT-SPIONs. All values reported are mean ± SEM (*n* = 6). [^*^ for p < 0.05, ^**^ for p < 0.01 and ^***^ for p < 0.001]. QT, quercetin; h, hours

There are numerous analytical techniques suitable for bioavailability study and measuring of QT in organic fluids. HPLC–UV, HPLC-FD (fluorometric detection), HPLC–ED (electrochemical detection) and GC–MS method have been used so far [37–40]. In this study, we used HPLC–UV which is adequate specificity and simplicity for quantifying of QT in plasma and brain tissue. The standard curve for quercetin was linear in the range of 0.05–0.6 μg/ml, which could be described by the regression equation: Y = 95215X + 118 (*r* = 0.9976) for quercetin where Y was the peak area of the analyte, and X was the concentration in μg/ml (Fig. 4). Figure 4 shows the concentration-time profiles of an individual dosage of 100 mg/kg of free QT and QT-SPIONs in the plasma. The maximum plasma concentration of free QT solution (100 mg/kg) within 6 h after oral administration was 0.402 ± 0.133 μg/ml (Fig. 4b). This for 100 mg/kg in 3 h after oral administration of QT-SPIONs was 0.137 ± 0.029 μg/ml (Fig. 4c). The maximum plasma concentration of free QT was significantly higher (*p < 0.05*) in comparison to QT-SPIONs. While free QT disappears from plasma 6 h after administration, QT-SPIONs remains in plasma for 9 h (Fig. 4d). No quercetin was detected in control samples. Quercetin conjugated with SPIONs exhibited prolonged blood circulation compared to free ones.

### Detection of quercetin in brain

**Fig. 5:**
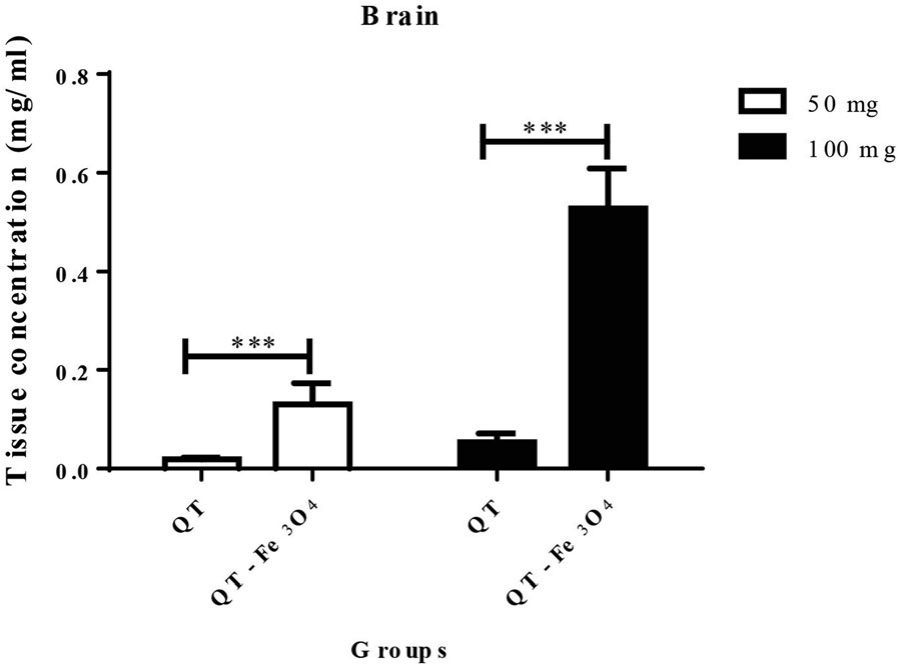
HPLC analyses for the biodistribution of quercetin in rat brain after oral administration of quercetin and quercetin conjugated Fe_3_O_4_ nanoparticles. QT, quercetin. All values reported are mean ± SEM (n = 6). [* for *p* < 0.05, ** for *p* < 0.01 and *** for *p* < 0.001]. QT, quercetin

HPLC was used to detect quercetin concentration in brain tissues. Figure 5 shows the quercetin concentration in brain tissue after treatment of animals with free QT, and QT-SPIONs over a time period of 7 days. The concentrations of QT in the brain for 50 mg/kg, and 100 mg/kg were 0.019 ± 0.003 and 0.053 ± 0.018 μg/ml, respectively. Nevertheless, the concentration of quercetin in the brain of rats treated with 50 mg/kg and 100 mg/kg of QT- Fe_3_O_4_ nanoparticles was 0.13 ± 0.04 and 0.52 ± 0.08 respectively. Brains of the rats were fed with QT-SPIONs shows higher concentrations of QT when compared with those that were fed with free QT.

### Detection of iron in plasma and brain

**Fig. 6:**
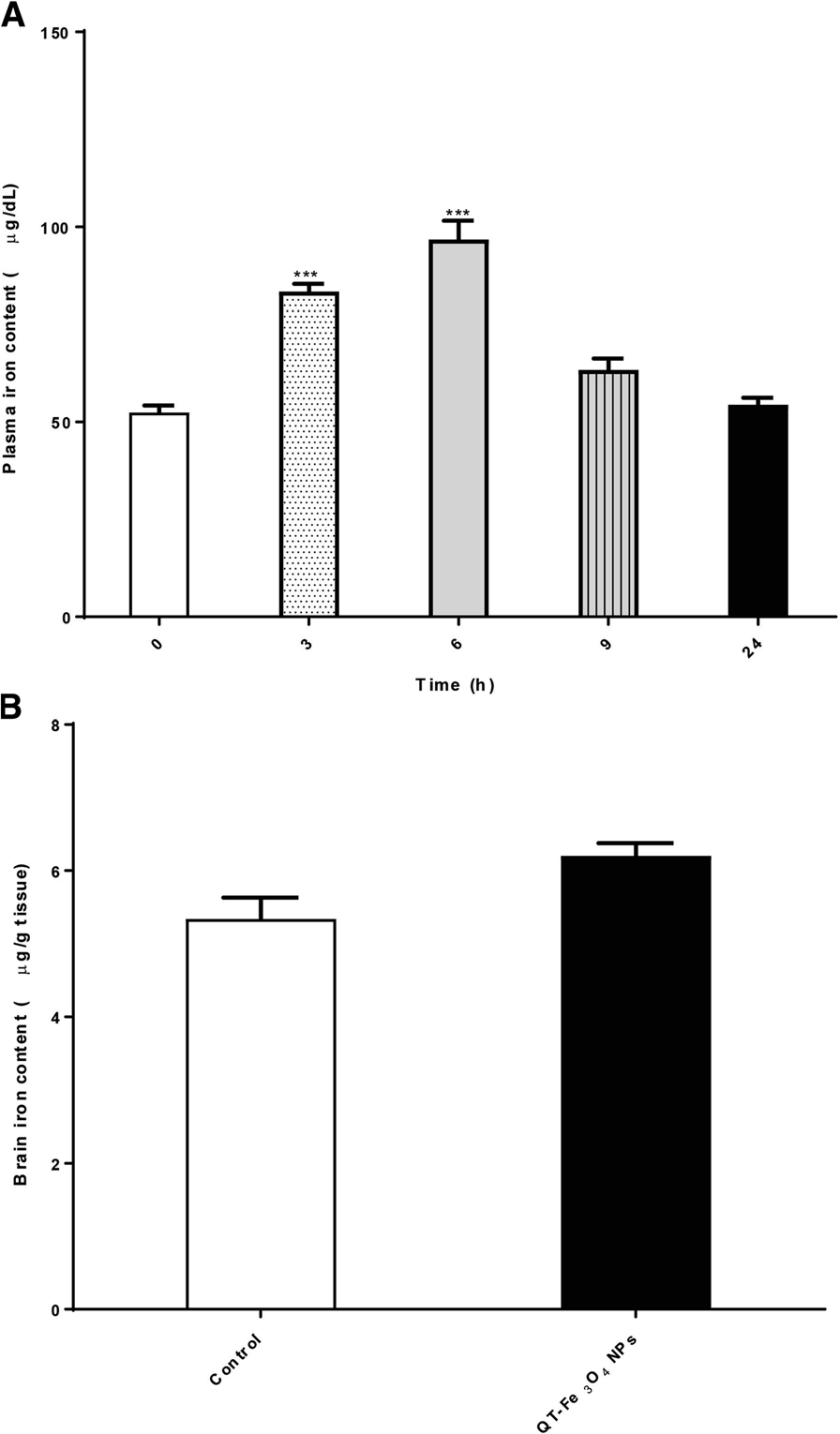
Total iron concentration in plasma and brain. **a** analyses for the identification of total iron in rat plasma after oral administration of quercetin conjugated Fe_3_O_4_ nanoparticles (100 mg/kg) in Wistar rats. **b** analyses for the determination of iron content in rat brain after oral administration of quercetin conjugated Fe_3_O_4_ nanoparticles (100 mg/kg.) All values reported are mean ± SEM (n = 3). QT, quercetin; h, hours. [*** for *p* < 0.001]. QT, quercetin

The iron accumulations in the plasma at 0, 3, 6, 9, and 24 h after oral treatment of 100 mg/kg of QT-SPIONs were quantified by ICP-OES. For the brain tissue, this was down after 24 h of administration of QT-SPION. In control rats, the iron content of the plasma was 52.4 ± 1.8 μg/dL. Plasma iron concentrations in rats after 3, 6, 9, and 24 h were 83.3 ± 2.02, 96.6 ± 4.9, 63.3 ± 2.9, and 54.3 ± 1.7 μg/dL respectively. A time course showed that total serum iron levels were low at hour 3, increased significantly at hour 6, and decreased at hour 9 (Fig. 6a). Brain iron contents were 5.3 ± 0.3 μg/g tissue in control rats and 6.2 ± 0.1 μg/g tissue in rats 24 h after superparamagnetic iron oxide nanoparticles administration, which is less than the iron content in the plasma (Fig. 6b). There is no significant difference in brain iron contents in control and treated rats.

### Histopathologic alterations

**Fig. 7:**
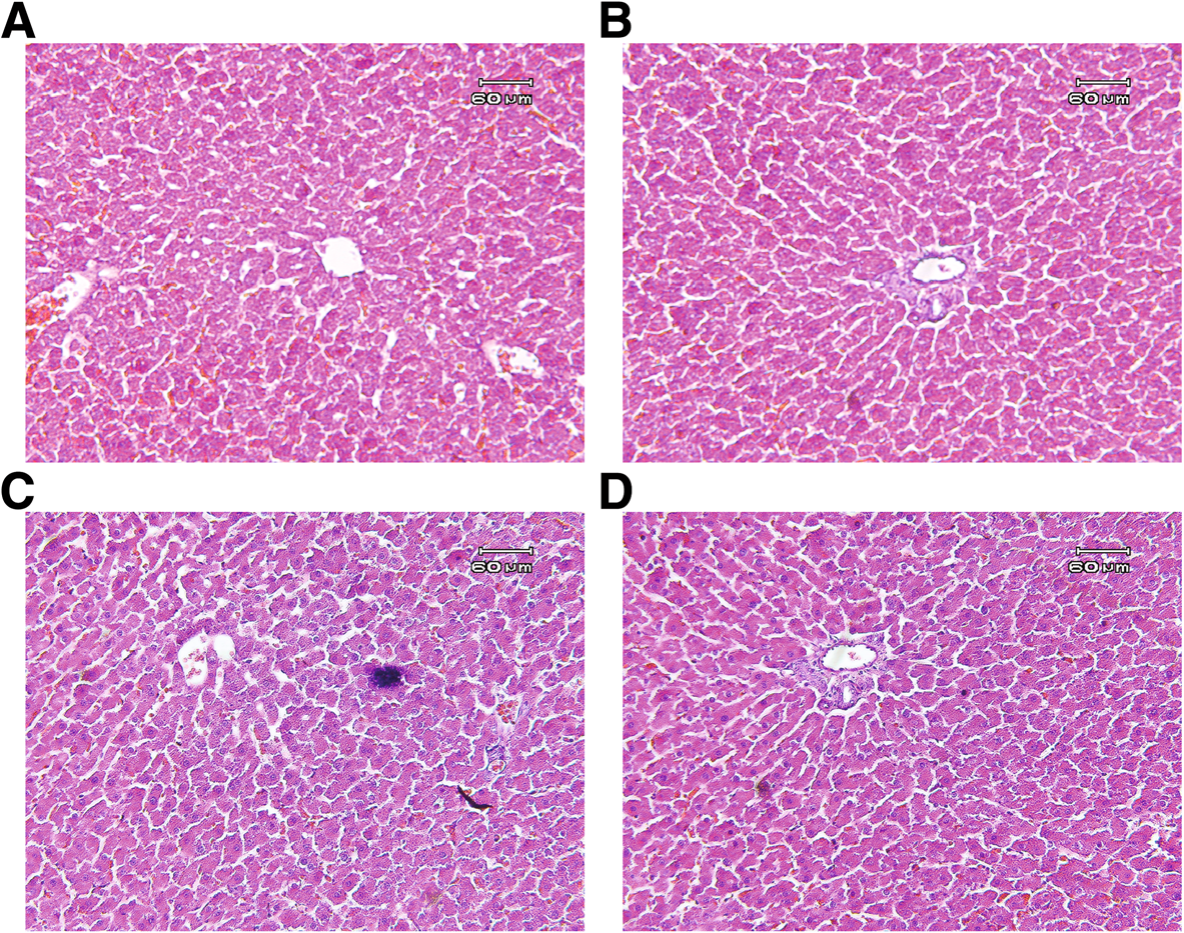
Liver histology of the control and experimental rats. The figure shows the effect of Fe_3_O_4_NP, QT, QT-SPIONs (at a dose of 100 mg/kg, and 7 days) on histology of liver of rats. **a** control; **b** free quercetin (QT); **c** SPIONs; **d** QT-SPIONs. The histology shows the normal liver architecture for all treatments (H & E staining)

Histology of liver tissues is shown in Fig. 7. Hepatic sections of control rats showed a regular lobular pattern with a central vein, slit-like sinusoids, and prominent nuclei. No histological alterations were detected in the liver tissue of treated rats.

## Discussion

Quercetin has high antioxidant potential in comparison with a number of flavonoids and could be used to treat or prevent the development and progression of neurodegenerative diseases [28]. Quercetin in combination with ascorbic acid decreases glutathione (GSH) levels in the neural cells. These results were interpreted as a confirmation of the effect of quercetin on oxidative stress [41]. Oyama and colleagues as the first group of investigators who explored the effect of quercetin contained *Ginkgo biloba* extract on the neurons suffered from oxidative stress caused by hydrogen peroxide found that quercetin reduces the oxidative metabolism in a dose dependent way [42]. Naidu et al., studied the effect of quercetin on the lipid peroxidation and found that Superoxide Dismutase (SOD), and catalase enzymes levels decreased after the administration of quercetin. Hence they suggested that probable action mechanism of quercetin in order to inhibit activities of oxidases, and Nitric Oxide Synthase (NOS) enzymes include scavenging of free radicals and chelating the metal ions such as iron and copper [43]. Studying the effect of quercetin and its glycoside metabolite on the apoptosis rate of glioma cell line revealed that quercetin treatment inhibits cell death induced by oxidative stress [44]. Since the antioxidant effect of quercetin was verified in a number of studies, a new step initiated through studying the effect of this compound on the neurodegenerative diseases. Investigation of the neuroprotective effect of quercetin on the cognitive impairment induced by D-galactose was also in consistent with the previous studies. Therefore, the activity of SOD increased and the concentration of Ca^2+^ was controlled in the brain tissue that all indicate a more complexed action mechanism for quercetin [45]. The main limitation of quercetin is low solubility and bioavailability.

The current study describes a simple precipitation method to prepare the quercetin conjugated with superparamagnetic nanoparticles and investigates the bioavailability of quercetin using this approach in the intact rat brain. Recent studies designated successful loading of quercetin onto magnetic nanoparticles [35, 36, 46]. The novel QT-Fe_3_O_4_ nanoparticles described in this work have the desired characteristics to be used as a carrier system. The results of the physicochemical characterization of QT-SPIONs are in line with a previous report by Kumar et al. [35]. Results of this study showed that the quercetin was effectively conjugated to the dextran coated superparamagnetic iron oxide nanoparticles (Fig. 1). In vivo study showed that Fe_3_O_4_ nanoparticles are relatively safe because they leave the body quickly and therefore, in vital organs no accumulation of nanoparticles occur [1, 47]. In our study, no mortalities were detected in rats fed with Fe_3_O_4_ NPs and QT-SPIONs. In a study [48], the toxicity of Fe_3_O_4_ ferrofluid (6 mg/rat) in rats were evaluated. No changes in mortality were detected in this study. Histopathological results showed at least during the period the rats were treated with NPs, QT-SPIONs and free QT no toxic effect was observed. As we previously reported rats fed with NPs showed a significant weight loss whereas those that fed QT-SPION didn’t [49].

By entering nanoparticles into the gastrointestinal tract through the oral route, the NPs can be absorbed through the epithelium and enter the blood circulation. This route for NPs administration is the most useful one especially in the case of chronic diseases. Nanoparticles help to overcome limitations such as varied-range pH, enzymes degradation and poor solubility, penetrability, and dissolution in the gastrointestinal system. The small particle size of NPs increases the contact with the epithelial surfaces and leads in more uptake of the particles [50]. Nevertheless, several factors such as particle size, nanoparticle stability, surface charge, the intestinal contents, and the residence time of nanoparticles at the absorption site affect the absorption and bioavailability of the nanoparticles [51].

The size, composition and surface hydrophilicity of a drug affect its distribution in the body. The major site of metabolism of particles is the liver, where most of the metabolizing enzymes are found. The metabolism process involves adding or modifying a functional group on the surface of the drug or involves conjugation of the drug with endogenous groups. Metabolism of nanoparticles is a bit more complicated because of the difference in its structure. In the liver, the ligands associated with nanoparticles are metabolized along with them. Nevertheless, the precise pathway of NPs metabolism is unknown yet. The optimum demand for nanoparticles is readily clearance from the body without causing any toxic effects. The particle material, size, shape, surface chemistry, and charge of NPs determine their modifications. In summary, the size, shape, structure, and coating [52] have a significant effect on the cellular uptake, cytotoxicity, distribution and clearance of INOPs.

The drug release behavior of quercetin conjugated to the dextran-coated SPIONs was investigated using a dialysis membrane in basic medium. Our results showed that almost 60% of the drug released within 8 h of dialysis (Fig. 3). However, the quercetin release from SPIONs was slow and controlled in the basic release medium. It is clear that an efficient drug delivery system such as SPIONs, need to have the capability to release their drug cargo at an optimum amount. Nevertheless, it has been reported that a large part of the drug cargo is rapidly released from the in vivo administration of SPIONs. This phenomenon is called as a burst effect. Therefore, minor quantities of the drug reach the specific site after drug treatment. Different strategies such as using different coating [53], mesoporous structure [54] have been used to reduce the burst effect. Finally, the results of drug release in the current study are in line with findings of quercetin biodistribution in plasma.

In the next step, rats were fed with free quercetin and quercetin conjugated Fe_3_O_4_ nanoparticles to compare the bioavailability of the QT and QT in conjugated with Fe_3_O_4_ NPs in the plasma and the brain of healthy rats using HPLC assay. The plasma concentrations of the QT and QT-SPIONs deteriorated quickly after 6 h. The clearance rate of QT from the blood was significantly higher for the free QT than QT in conjugated with Fe_3_O_4_ NPs. This phenomenon could be due to quick distribution and metabolization of the free QT. Alongside with this study, Manach et al. reported rapid metabolization of the quercetin by the intestinal enzymes [55]. The plasma concentration of QT-SPIONs displayed an enhancement in oral bioavailability. This reveals more constant release of QT from NPs in comparison to free QT. Alternatively, the small size of QT-SPIONs causes a high quantity of quercetin in plasma. Additionally, dextran coating of Fe_3_O_4_ NPs may aid in holding the QT in the blood circulation for a long period [56, 57]. Jain and colleagues in 2013 reported that using self-emulsifying drug delivery systems could increase the plasma concentration of the quercetin in comparison with free QT solution [58]. Also, it has been reported that the encapsulation of the quercetin in polymeric nanoparticles increased the oral bioavailability of QT when compared with free QT [59]. Other research groups have reported coupling NPs with QT improved oral bioavailability of the quercetin [60–64].

It should be reminded that in our study the loading of the QT in QT-SPIONs is 42% which means QT significantly is lower than that of free QT because only a part of QT- Fe_3_O_4_ is quercetin. Furthermore, the concentration of QT in the brain of rats fed with 50 mg /kg QT- Fe_3_O_4_ NPs and 100 mg /kg QT- Fe_3_O_4_ NPs were significantly higher (*P*, 0.05) than rats treated with free QT (Fig. 5).

The concentration of quercetin in the brain delivered by Fe_3_O_4_ nanoparticles at the concentration of 50 and 100 mg/kg were about 7 and 10-folds higher respectively in comparison with the free quercetin. It designates that Fe_3_O_4_ nanoparticles increased the bioavailability of quercetin. From this observation, QT-SPIONs enhance the biodistribution of quercetin into the brain. Thus, because of the Fe_3_O_4_ nanoparticles, the levels of QT are significantly higher than the free quercetin in the plasma and the brain.

One advantage of nanoparticle drug delivery systems in comparison to the free drugs is mostly due to elongated blood circulation. In our study, the concentration of quercetin in the blood increased significantly. Prolonged quercetin circulation in the blood may enhance drug capability to interact with particular carriers or receptors localized inside of the blood-brain barrier endothelial cells and, accordingly, to passage the blood-brain barrier. It has been shown that receptor-mediated transport is the most effective carrying mechanism that occurs on the luminal side of the brain capillary endothelium.

It may be questioned whether quercetin enhancement of the brain tissues is because of the blood circulation in the brain due to lack of blood perfusion. As shown quercetin was almost disappeared completely from the blood circulation after nine hours of treatment. QT was determined in the brain after 24 h of the QT administration on day 7 of treatment. Therefore, the amount of quercetin in the brain couldn’t be due to the existence of the QT in the blood vessels.

The blood-brain barrier (BBB) is contained of a contiguous layer of endothelial cells connected by tight junctions. These tight junctions are almost 100 times tighter than junctions of other capillary endothelium [65]. Therefore, the barrier allowing lipid-soluble molecules transport across the membrane while hydrophilic solutes exhibit insignificant permeation [66]. To overcome the barrier one approach is altering barrier integrity or characteristics, and another approach is changing the characteristics of the drug. Since there are risks related to changing the permeability of the BBB, efforts have been made to modify drugs to more readily cross the barrier.

To cross the BBB, the size and weight of nanoparticles should be in a certain range [67]. So that 20 to 100 nm is the optimum size in order to pass through BBB [68] and consequently smaller size of nanoparticles is associated with more efficient drug delivery. Nanoparticles by passive diffusion and receptor-mediated endocytosis can cross the BBB.

To verify whether SPIONs could cross the BBB, we measured the amount of iron contents of the plasma and brain. The results showed that superparamagnetic iron oxide nanoparticles penetrated from the digestive system to the blood circulation. Considering time points iron returned to the normal levels 9 h after SPIONs treatment. The profile of plasma iron contents is very similar to the profile of the plasma quercetin concentration. However, the iron contents of the brain were not significantly higher in the brain of treated rats than control rats. In line with the reported study [9], the results of the current research revealed that SPIONs couldn’t cross the BBB [69]. This might be due to the existence of various proteins, for instance, multidrug resistance protein and P-glycoprotein on the BBB cells function as efflux proteins [70]. Some of these proteins such as multidrug resistance proteins are ATP-dependent and drive numerous external nanoparticles out of cells. However, magnetic nanoparticles could increase penetration of the BBB. The sensitivity of brain cells to these nanoparticles is higher than other organs cells [71, 72].

SPIONs can carry the loaded drug to the desired target area using an external magnetic field and at the same time tracking the biodistribution of the particles [73]. This approach as a modern technology is known as theragnositc (therapeutic and diagnostic). This method could reduce the side effects and the required dosage of the drugs [74–77]. Use of the exterior magnetic field facilitates uptake of Fe_3_O_4_ NPs by brain cells [78]. Therefore, nanoparticles-based drug carriers are promising to improve the special brain drug delivery system. However, the external magnetic field may increase NPs aggregation [79].

Although the research has reached to its aims, due to time, pricing and ethical concerns, there were some unavoidable limitations. First, because of the time limit, this study was conducted only for one week. Therefore, to generalize the results, the research should be done in a longer time. Second, due to the unavailability of funds, the current study has not investigated the lower or higher dosage of the treatment or has not included sex of the rats, cellular study and so on. Finally, measurement of quercetin and histological assessment have been done only for plasma and the brain, it is ideal to do these for other tissues of the body. To avoid the complexity of the subject it is necessary to study these limitations in separate studies.

## Conclusions

In the current study, the capability of SPIONs covered with dextran was investigated for bioavailability improvement of the quercetin, in the brain of a healthy rat model. Neither QT nor QT-SPIONs remain in plasma more than of 9 h. The plasma concentrations of QT in rats treated with QT- SPIONs were higher than those of free quercetin. Therefore, it can be concluded that SPIONs coated with dextran enhance delivery of the poorly water soluble quercetin to the brain. Accordingly, this delivery system may be a promising feature in the clinical application. Also, these results propose a potential effective strategy for regulating the biodistribution of QT-SPIONs in the brain by means of an external magnetic field. In future nanoparticle carrier systems may have a significant role in the treatment of the neurological disorders.

## Abbreviations

BBB: Blood–brain barrier
CNS: Central nervous system
FTIR: Fourier transformed infrared spectrometer
HPLC: high-performance liquid chromatography
NPs: Nanoparticles
QT: Quercetin
SEM: Scanning electron microscope
SPIONs: Superparamagnetic iron oxide nanoparticles
XRD: X-ray diffractometer

## References

[1] Barreto A, Santiago V, Mazzetto S, Denardin JC, Lavín R, Mele G (2011). Magnetic nanoparticles for a new drug delivery system to control quercetin releasing for cancer chemotherapy. J Nanopart Res.

[2] Chowdhury P, Nagesh PK, Kumar S, Jaggi M, Chauhan SC, Yallapu MM. Pluronic nanotechnology for overcoming drug resistance. In: Bioactivity of Engineered Nanoparticles: Springer; 2017. p. 207–37.

[3] Valdiglesias V, Kiliç G, Costa C, Fernández-Bertólez N, Pásaro E, Teixeira JP (2015). Effects of iron oxide nanoparticles: cytotoxicity, genotoxicity, developmental toxicity, and neurotoxicity. Environ Mol Mutagen.

[4] Vinzant N, Scholl JL, Wu C-M, Kindle T, Koodali R, Forster GL (2017). Iron oxide nanoparticle delivery of peptides to the brain: reversal of anxiety during drug withdrawal. Front Neurosci.

[5] Pardridge WM (2002). Why is the global CNS pharmaceutical market so under-penetrated?. Drug Discov Today.

[6] Zheng M, Tao W, Zou Y, Farokhzad OC, Shi B (2018). Nanotechnology-based strategies for siRNA brain delivery for disease therapy. Trends in biotechnology.

[7] Durães F, Pinto M, Sousa E (2018). Old drugs as new treatments for neurodegenerative diseases. Pharmaceuticals.

[8] Neuwelt E, Abbott NJ, Abrey L, Banks WA, Blakley B, Davis T (2008). Strategies to advance translational research into brain barriers. Lancet Neurol.

[9] Huang Y, Zhang B, Xie S, Yang B, Xu Q, Tan J (2016). Superparamagnetic Iron oxide nanoparticles modified with tween 80 pass through the intact blood–brain barrier in rats under magnetic field. ACS Appl Mater Interfaces.

[10] Kiviniemi V, Korhonen V, Kortelainen J, Rytky S, Keinänen T, Tuovinen T (2017). Real-time monitoring of human blood-brain barrier disruption. PLoS One.

[11] Chauhan NB (2002). Trafficking of intracerebroventricularly injected antisense oligonucleotides in the mouse brain. Antisense Nucleic Acid Drug Dev.

[12] Westphal M, Ram Z, Riddle V, Hilt D, Bortey E, Group ECotGS (2006). Gliadel® wafer in initial surgery for malignant glioma: long-term follow-up of a multicenter controlled trial. Acta Neurochir.

[13] Abbott NJ, Romero IA (1996). Transporting therapeutics across the blood-brain barrier. Mol Med Today.

[14] Oldendorf WH (1970). Measurement of brain uptake of radiolabeled substances using a tritiated water internal standard. Brain Res.

[15] Wu D, Pardridge WM (1998). Pharmacokinetics and blood-brain barrier transport of an anti-transferrin receptor monoclonal antibody (OX26) in rats after chronic treatment with the antibody. Drug Metab Dispos.

[16] Illum L (2003). Nasal drug delivery—possibilities, problems and solutions. J Control Release.

[17] Kumari A, Yadav SK, Pakade YB, Singh B, Yadav SC (2010). Development of biodegradable nanoparticles for delivery of quercetin. Colloids Surf B: Biointerfaces.

[18] Wu T-H, Yen F-L, Lin L-T, Tsai T-R, Lin C-C, Cham T-M (2008). Preparation, physicochemical characterization, and antioxidant effects of quercetin nanoparticles. Int J Pharm.

[19] Bagad M, Khan ZA (2015). Poly (n-butylcyanoacrylate) nanoparticles for oral delivery of quercetin: preparation, characterization, and pharmacokinetics and biodistribution studies in Wistar rats. Int J Nanomedicine.

[20] Stewart LK, Soileau JL, Ribnicky D, Wang ZQ, Raskin I, Poulev A (2008). Quercetin transiently increases energy expenditure but persistently decreases circulating markers of inflammation in C57BL/6J mice fed a high-fat diet. Metabolism.

[21] Ramos FA, Takaishi Y, Shirotori M, Kawaguchi Y, Tsuchiya K, Shibata H (2006). Antibacterial and antioxidant activities of quercetin oxidation products from yellow onion (Allium cepa) skin. J Agric Food Chem.

[22] Chakraborty S, Stalin S, Das N, Choudhury ST, Ghosh S, Swarnakar S (2012). The use of nano-quercetin to arrest mitochondrial damage and MMP-9 upregulation during prevention of gastric inflammation induced by ethanol in rat. Biomaterials.

[23] Choi H-J, Kim J-H, Lee C-H, Ahn Y-J, Song J-H, Baek S-H (2009). Antiviral activity of quercetin 7-rhamnoside against porcine epidemic diarrhea virus. Antivir Res.

[24] Duarte J, Pérez-Vizcaíno F, Zarzuelo A, Jiménez J, Tamargo J (1993). Vasodilator effects of quercetin in isolated rat vascular smooth muscle. Eur J Pharmacol.

[25] Davis JM, Murphy EA, Carmichael MD, Davis B (2009). Quercetin increases brain and muscle mitochondrial biogenesis and exercise tolerance. Am J Phys Regul Integr Comp Phys.

[26] Sandeep M, Nandini C (2017). Influence of quercetin, naringenin and berberine on glucose transporters and insulin signalling molecules in brain of streptozotocin-induced diabetic rats. Biomed Pharmacother.

[27] Ossola B, Kääriäinen TM, Männistö PT (2009). The multiple faces of quercetin in neuroprotection. Expert Opin Drug Saf.

[28] Dajas F (2012). Life or death: neuroprotective and anticancer effects of quercetin. J Ethnopharmacol.

[29] Dajas F, Juan Andres A-C, Florencia A, Carolina E, Felicia R-M (2013). Neuroprotective actions of flavones and flavonols: mechanisms and relationship to flavonoid structural features. Cent Nerv Syst Agents Med Chem.

[30] Nichols M, Zhang J, Polster B, Elustondo P, Thirumaran A, Pavlov E (2015). Synergistic neuroprotection by epicatechin and quercetin: activation of convergent mitochondrial signaling pathways. Neuroscience.

[31] Lee Yang-ja, Bernstock Joshua D., Nagaraja Nandakumar, Ko Brian, Hallenbeck John M. (2016). Global SUMOylation facilitates the multimodal neuroprotection afforded by quercetin against the deleterious effects of oxygen/glucose deprivation and the restoration of oxygen/glucose. Journal of Neurochemistry.

[32] Rivera F, Urbanavicius J, Gervaz E, Morquio A, Dajas F (2004). Some aspects of thein vivo neuroprotective capacity of flavonoids: bioavailability and structure-activity relationship. Neurotox Res.

[33] Blasina F, Vaamonde L, Silvera F, Tedesco AC, Dajas F (2015). Intravenous nanosomes of quercetin improve brain function and hemodynamic instability after severe hypoxia in newborn piglets. Neurochem Int.

[34] Galho A, Cordeiro M, Ribeiro S, Marques M, Antunes M, Luz D (2016). Protective role of free and quercetin-loaded nanoemulsion against damage induced by intracerebral haemorrhage in rats. Nanotechnology.

[35] Kumar SR, Priyatharshni S, Babu V, Mangalaraj D, Viswanathan C, Kannan S (2014). Quercetin conjugated superparamagnetic magnetite nanoparticles for in-vitro analysis of breast cancer cell lines for chemotherapy applications. J Colloid Interface Sci.

[36] Akal Z, Alpsoy L, Baykal A (2016). Superparamagnetic iron oxide conjugated with folic acid and carboxylated quercetin for chemotherapy applications. Ceram Int.

[37] Bongartz D, Hesse A (1995). Selective extraction of quercetrin in vegetable drugs and urine by off-line coupling of boronic acid affinity chromatography and high-performance liquid chromatography. J Chromatogr B Biomed Sci Appl.

[38] Erlund I, Alfthan G, Siren H, Ariniemi K, Aro A (1999). Validated method for the quantitation of quercetin from human plasma using high-performance liquid chromatography with electrochemical detection. J Chromatogr B Biomed Sci Appl.

[39] Yochum L, Kushi LH, Meyer K, Folsom AR (1999). Dietary flavonoid intake and risk of cardiovascular disease in postmenopausal women. Am J Epidemiol.

[40] Soleas G, Yan J, Goldberg D (2001). Ultrasensitive assay for three polyphenols (catechin, quercetin and resveratrol) and their conjugates in biological fluids utilizing gas chromatography with mass selective detection. J Chromatogr B Biomed Sci Appl.

[41] Skaper SD, Fabris M, Ferrari V, Dalle Carbonare M, Leon A (1997). Quercetin protects cutaneous tissue-associated cell types including sensory neurons from oxidative stress induced by glutathione depletion: cooperative effects of ascorbic acid. Free Radic Biol Med.

[42] Oyama Y, Fuchs PA, Katayama N, Noda K (1994). Myricetin and quercetin, the flavonoid constituents ofGinkgo biloba extract, greatly reduce oxidative metabolism in both resting and Ca2+−loaded brain neurons. Brain Res.

[43] Naidu PS, Singh A, Kulkarni SK (2003). Quercetin, a bioflavonoid, attenuates haloperidol-induced orofacial dyskinesia. Neuropharmacology.

[44] Le Nest G, Caille O, Woudstra M, Roche S, Guerlesquin F, Lexa D (2004). Zn–polyphenol chelation: complexes with quercetin,(+)-catechin, and derivatives: I optical and NMR studies. Inorg Chim Acta.

[45] LU J, ZHENG Y, LUO L, WU D, SUN D, FENG Y (2006). Quercetin reverses d-galactose induced neurotoxicity in mouse brain. Behavioural Brain Research.

[46] Malekzadeh AM, Ramazani A, Rezaei SJT, Niknejad H. Design and construction of multifunctional hyperbranched polymers coated magnetite nanoparticles for both targeting magnetic resonance imaging and cancer therapy. J. Colloid Interface Sci. 2017;490:64–73. 10.1016/j.jcis.2016.11.014 27870961

[47] Boyer C, Whittaker MR, Bulmus V, Liu J, Davis TP (2010). The design and utility of polymer-stabilized iron-oxide nanoparticles for nanomedicine applications. NPG Asia Mat.

[48] Hong R, Feng B, Chen L, Liu G, Li H, Zheng Y (2008). Synthesis, characterization and MRI application of dextran-coated Fe 3 O 4 magnetic nanoparticles. Biochem Eng J.

[49] Najafabadi RE, Kazemipour N, Esmaeili A, Beheshti S, Nazifi S. Quercetin prevents body weight loss due to the using of superparamagnetic Iron oxide nanoparticles in rat. Adv Biomed Res. 2018;7. 10.4103/abr.abr_141_17 PMC5812102 29456979

[50] Pridgen EM, Alexis F, Farokhzad OC (2015). Polymeric nanoparticle drug delivery technologies for oral delivery applications. Expert Opin Drug Deliv.

[51] Alai M, Lin WJ (2015). Application of nanoparticles for oral delivery of acid-labile lansoprazole in the treatment of gastric ulcer: in vitro and in vivo evaluations. Int J Nanomedicine.

[52] Feng Q, Liu Y, Huang J, Chen K, Huang J, Xiao K (2018). Uptake, distribution, clearance, and toxicity of iron oxide nanoparticles with different sizes and coatings. Sci Rep.

[53] Mahmoudi M, Sant S, Wang B, Laurent S, Sen T (2011). Superparamagnetic iron oxide nanoparticles (SPIONs): development, surface modification and applications in chemotherapy. Adv Drug Deliv Rev.

[54] Guo S, Li D, Zhang L, Li J, Wang E (2009). Monodisperse mesoporous superparamagnetic single-crystal magnetite nanoparticles for drug delivery. Biomaterials.

[55] Manach C, Texier O, Morand C, Crespy V, Régérat F, Demigné C (1999). Comparison of the bioavailability of quercetin and catechin in rats. Free Radic Biol Med.

[56] Sun M, Gao Y, Guo C, Cao F, Song Z, Xi Y (2010). Enhancement of transport of curcumin to brain in mice by poly (n-butylcyanoacrylate) nanoparticle. J Nanopart Res.

[57] Gulyaev AE, Gelperina SE, Skidan IN, Antropov AS, Kivman GY, Kreuter J (1999). Significant transport of doxorubicin into the brain with polysorbate 80-coated nanoparticles. Pharm Res.

[58] Jain S, Jain AK, Pohekar M, Thanki K (2013). Novel self-emulsifying formulation of quercetin for improved in vivo antioxidant potential: implications for drug-induced cardiotoxicity and nephrotoxicity. Free Radic Biol Med.

[59] Jain AK, Thanki K, Jain S (2013). Co-encapsulation of tamoxifen and quercetin in polymeric nanoparticles: implications on oral bioavailability, antitumor efficacy, and drug-induced toxicity. Mol Pharm.

[60] Dhawan S, Kapil R, Singh B (2011). Formulation development and systematic optimization of solid lipid nanoparticles of quercetin for improved brain delivery. J Pharm Pharmacol.

[61] Zhao L, Liu A, Sun M, Gu J, Wang H, Wang S (2011). Enhancement of oral bioavailability of puerarin by polybutylcyanoacrylate nanoparticles. J Nanomater.

[62] Yordanov G (2012). Influence of the preparation method on the physicochemical properties of econazole-loaded poly (butyl cyanoacrylate) colloidal nanoparticles. Colloids Surf A Physicochem Eng Asp.

[63] Tian X-h, Lin X-N, Wei F, Feng W, Huang Z-C, Wang P (2011). Enhanced brain targeting of temozolomide in polysorbate-80 coated polybutylcyanoacrylate nanoparticles. Int J Nanomedicine.

[64] Ghosh A, Mandal AK, Sarkar S, Panda S, Das N (2009). Nanoencapsulation of quercetin enhances its dietary efficacy in combating arsenic-induced oxidative damage in liver and brain of rats. Life Sci.

[65] Butt AM, Jones HC, Abbott NJ (1990). Electrical resistance across the blood-brain barrier in anaesthetized rats: a developmental study. J Physiol.

[66] McConnell H (2018). Ferumoxytol Iron oxide nanoparticles as a magnetic resonance contrast agent in central nervous system lesions.

[67] Talegaonkar S, Mishra P (2004). Intranasal delivery: an approach to bypass the blood brain barrier. Indian J Pharmacol.

[68] Jo DH, Kim JH, Lee TG, Kim JH (2015). Size, surface charge, and shape determine therapeutic effects of nanoparticles on brain and retinal diseases. Nanomedicine.

[69] Rousseau V, Denizot B, Pouliquen D, Jallet P, Le Jeune J (1997). Investigation of blood-brain barrier permeability to magnetite-dextran nanoparticles (MD3) after osmotic disruption in rats. MAGMA.

[70] Taccola C, Cartot-Cotton S, Valente D, Barneoud P, Aubert C, Boutet V (2018). High brain distribution of a new central nervous system drug candidate despite its P-glycoprotein-mediated efflux at the mouse blood-brain barrier. Eur J Pharm Sci.

[71] Laurent S, Burtea C, Thirifays C, Häfeli UO, Mahmoudi M (2012). Crucial ignored parameters on nanotoxicology: the importance of toxicity assay modifications and “cell vision”. PLoS One.

[72] Mahmoudi M, Laurent S, Shokrgozar MA, Hosseinkhani M (2011). Toxicity evaluations of superparamagnetic iron oxide nanoparticles: cell “vision” versus physicochemical properties of nanoparticles. ACS Nano.

[73] Shubayev VI, Pisanic TR, Jin S (2009). Magnetic nanoparticles for theragnostics. Adv Drug Deliv Rev.

[74] Neuberger Tobias, Schöpf Bernhard, Hofmann Heinrich, Hofmann Margarete, von Rechenberg Brigitte (2005). Superparamagnetic nanoparticles for biomedical applications: Possibilities and limitations of a new drug delivery system. Journal of Magnetism and Magnetic Materials.

[75] Lübbe AS, Alexiou C, Bergemann C (2001). Clinical applications of magnetic drug targeting. J Surg Res.

[76] Rudge S, Peterson C, Vessely C, Koda J, Stevens S, Catterall L (2001). Adsorption and desorption of chemotherapeutic drugs from a magnetically targeted carrier (MTC). J Control Release.

[77] Zanganeh S., Ho J.Q., Aieneravaie M., Erfanzadeh M., Pauliah M., Spitler R. (2018). Drug Delivery. Iron Oxide Nanoparticles for Biomedical Applications.

[78] Thomsen LB, Linemann T, Pondman KM, Lichota J, Kim KS, Pieters RJ (2013). Uptake and transport of superparamagnetic iron oxide nanoparticles through human brain capillary endothelial cells. ACS Chem Neurosci.

[79] Hamley I (2003). Nanotechnology with soft materials. Angew Chem Int Ed.

